# Metagenomic next-generation sequencing of bronchoalveolar lavage fluid from children with severe pneumonia in pediatric intensive care unit

**DOI:** 10.3389/fcimb.2023.1082925

**Published:** 2023-03-16

**Authors:** Caiyan Zhang, Tingyan Liu, Yixue Wang, Weiming Chen, Jing Liu, Jinhao Tao, Zhengzheng Zhang, Xuemei Zhu, Zhenyu Zhang, Meixiu Ming, Mingbang Wang, Guoping Lu, Gangfeng Yan

**Affiliations:** ^1^ Paediatric Intensive Care Unit, Children’s Hospital of Fudan University, Shanghai, China; ^2^ Shanghai Key Laboratory of Birth Defects, Division of Neonatology, Children’s Hospital of Fudan University, National Center for Children’s Health, Shanghai, China; ^3^ Microbiome Therapy Center, South China Hospital, Medical School, Shenzhen University, Shenzhen, China

**Keywords:** metagenomic next-generation sequencing, severe pneumonia, bronchoalveolar lavage fluid, Epstein–Barr virus, *Pneumocystis jirovecii*

## Abstract

**Background:**

Severe pneumonia due to lower respiratory tract infections (LRTIs) is a significant cause of morbidity and mortality in children. Noninfectious respiratory syndromes resembling LRTIs can complicate the diagnosis and may also make targeted therapy difficult because of the difficulty of identifying LRTI pathogens. In the present study, a highly sensitive metagenomic next-generation sequencing (mNGS) approach was used to characterize the microbiome of bronchoalveolar lavage fluid (BALF) in children with severe lower pneumonia and identify pathogenic microorganisms that may cause severe pneumonia. The purpose of this study was to use mNGS to explore the potential microbiomes of children with severe pneumonia in a PICU.

**Methods:**

We enrolled patients meeting diagnostic criteria for severe pneumonia admitted at PICU of the Children’s Hospital of Fudan University, China, from February 2018 to February 2020. In total, 126 BALF samples were collected, and mNGS was performed at the DNA and/or RNA level. The pathogenic microorganisms in BALF were identified and correlated with serological inflammatory indicators, lymphocyte subtypes, and clinical symptoms.

**Results:**

mNGS of BALF identified potentially pathogenic bacteria in children with severe pneumonia in the PICU. An increased BALF bacterial diversity index was positively correlated with serum inflammatory indicators and lymphocyte subtypes. Children with severe pneumonia in the PICU had the potential for coinfection with viruses including Epstein–Barr virus, *Cytomegalovirus*, and *Human betaherpesvirus 6B*, the abundance of which was positively correlated with immunodeficiency and pneumonia severity, suggesting that the virus may be reactivated in children in the PICU. There was also the potential for coinfection with fungal pathogens including *Pneumocystis jirovecii* and *Aspergillus fumigatus* in children with severe pneumonia in the PICU, and an increase in potentially pathogenic eukaryotic diversity in BALF was positively associated with the occurrence of death and sepsis.

**Conclusions:**

mNGS can be used for clinical microbiological testing of BALF samples from children in the PICU. Bacterial combined with viral or fungal infections may be present in the BALF of patients with severe pneumonia in the PICU. Viral or fungal infections are associated with greater disease severity and death.

**Importance**

During the COVID-19 pandemic, metagenomic next-generation sequencing (mNGS) of children admitted to the pediatric intensive care unit (PICU) with severe pneumonia has been an effective way to rapidly identify potentially pathogenic novel microorganisms.

In this retrospective study of bronchoalveolar lavage fluid samples from 126 children in the PICU, pathogenic microorganisms associated with severe pneumonia were identified by mNGS.

Pathogenic bacteria may be combined with viruses such as Epstein–Barr virus and *Cytomegalovirus*, and possibly fungi such as *Pneumocystis jirovecii* and *Aspergillus fumigatus*, in the bronchoalveolar lavage fluid of children with severe pneumonia admitted to the PICU.

## Background

The global COVID-19 pandemic is now in its third year. Even without COVID-19, however, pneumonia causes more deaths each year than any other type of infectious disease (Hansen et al., 2016). Accurate detection of pathogens and differentiation from background commensal organisms is essential to guide optimal antimicrobial therapy. Bronchoalveolar lavage fluid (BALF) is considered a specimen suitable for the detection of pathogens involved in respiratory tract infections (Friaza et al., 2010; Charlson et al., 2012; Willner et al., 2013; Seo et al., 2015). Metagenomic next-generation sequencing (mNGS) can be used to assess the microbial composition of clinical samples without culture, improving the sensitivity of pathogen detection and providing guidance for clinical practice (Gu et al., 2019). mNGS of BALF samples can identify pathogenic agents and improve treatment precision. Leo et al. (Leo et al., 2017) found that a protocol based on sequential lysis of human and bacterial cells or mechanical disruption of all cells could be used to extract DNA from BALF samples and perform mNGS assays. Fang et al. (Fang et al., 2018) performed mNGS on blood, BALF, and cerebrospinal fluid samples from an adult patient with severe pneumonia with unexplained fever and subsequent encephalitis, and they detected high loads of *human adenovirus B55*. The patient was treated with intravenous ribavirin and cleared the virus after 26 days, and mNGS testing was performed to diagnose unexpected herpes simplex virus 1 encephalitis during hospitalization, leading to timely treatment (Fang et al., 2018). Qi et al. (Qi et al., 2019) used mNGS on BALF samples from hospitalized patients with suspected ventilator-associated pneumonia. mNGS detected new pathogens in addition to those obtained by culture methods, while potential pathogens, including bacterial, fungal, and viral organisms, were detected by mNGS in 89% (40 of 45) culture-negative samples (Qi et al., 2019). Li et al. (Li et al., 2020a) performed mNGS on 35 BALF samples from 32 adults with respiratory failure. They found that the diagnostic sensitivity of mNGS was 88.89% and specificity was 74.07% compared with the culture method, with a concordance rate of 77.78% between the two methods; additionally, the diagnostic sensitivity of mNGS was 77.78% and specificity was 70.00% compared with the smear method and polymerase chain reaction. The diagnostic sensitivity of the mNGS findings led to a change in the treatment strategy in 11 of 32 (34.4%) patients (Li et al., 2020a).

The results of BALF mNGS testing in patients with severe respiratory infections have been shown to be similar to those of transbronchial lung biopsy (TBLB) and transtracheal aspiration. Liu et al. (Liu et al., 2019) compared mNGS testing on BALF samples and TBLB tissue samples from patients with peripheral lung infections. Whereas the specificity of mNGS was lower for BALF than for TBLB tissue, the sensitivity of mNGS was higher for BALF than for TBLB tissue, and the most common infectious agents of the lung were *Pseudomonas aeruginosa*, *Klebsiella pneumoniae*, and *Acinetobacter baumannii* (Liu et al., 2019). Kalantar et al. (Kalantar et al., 2019) performed mNGS on 52 mini-BALF samples from adults with severe pneumonia and compared them with mNGS results from tracheal aspirate samples. They found that although there were significant differences between sample types in patients with non-infectious acute respiratory disease, there were significant similarities in the composition of samples from patients with bacterial pneumonia, whose microbial community was characterized as the main pathogen (Kalantar et al., 2019).

Studies of mNGS on BALF samples from patients with severe respiratory infections have generally been limited to adults; few reports have focused on children. Takeuchi et al. (Takeuchi et al., 2019) performed mNGS on BALF samples from 10 children with respiratory failure and detected significant bacterial or viral sequencing reads in 8 of the 10 patients. In addition, candidate pathogens were detected in three patients in whom no pathogens were identified by conventional methods. Moreover, the complete genome of *enterovirus D68* was identified in two patients, and phylogenetic analysis showed that both strains belonged to subtype B3, a common strain that has spread worldwide in recent years (Takeuchi et al., 2019). These findings indicate that mNGS can be used for comprehensive molecular diagnosis as well as for pathogen surveillance in BALF in patients with respiratory tract infections. The aim of this study was to analyze the microbiome characteristics and explore the potential pathogens of BALF from pediatric patients with severe respiratory infection.

## Methods

### Participant enrollment

The participants in this study were children treated in the pediatric intensive care unit (PICU) of the Children’s Hospital of Fudan University from February 2018 to February 2020. The inclusion criterion was a diagnosis of severe pneumonia according to the diagnostic criteria established by the World Health Organization (Bradley et al., 2011; Chisti et al., 2015; Williams et al., 2016). The primary diagnostic criteria were invasive mechanical ventilation, fluid-refractory shock, an urgent need for noninvasive positive-pressure ventilation, and hypoxemia requiring an FiO_2_ greater than the inhalation concentration or flow rate feasible within general care. The secondary criteria were an increased respiratory rate, PaO_2_/FiO_2_ ratio of < 250, multilobar infiltration, Pediatric Early Warning Score of > 6, altered mental status, hypotension, presence of effusion, comorbidities (e.g. immunosuppression, immunodeficiency), and unexplained metabolic acidosis. The exclusion criteria for this study were an age of < 28 days or > 18 years; noninfectious factors such as congenital heart disease, pulmonary edema, asthma, upper airway obstruction, or pulmonary cystic fibrosis; contraindications to fiberoptic bronchoscopy; severe cardiopulmonary dysfunction; and coagulopathy.

### BALF collection

With reference to the recommendations of the European Respiratory Society (de Blic et al., 2000), sedation and topical anesthesia were administered, and the patient’s lungs were lavaged using an age-appropriate pediatric flexible fiberoptic bronchoscope. The more severely diseased region in patients with diffuse lung disease or the right middle lobe was selected based on radiological findings or evidence from bronchoscopy. Warm saline (1 mL/kg body weight, maximum 20 mL per fraction) was dripped into the selected lung lobes, and at least 40% of the fluid was recovered by mechanical suction using a pressure of approximately 50 to 100 mmHg.

### Serum inflammatory markers

Venous blood (2 mL) was collected and stored at room temperature with heparin anticoagulation to complete the assay for serum lipopolysaccharide (LPS), procalcitonin (PCT), C-reactive protein (CRP), interleukin 6 (IL-6), and other indices. The specific processes are detailed in the **Supplementary Appendix**.

### Lymphocyte subsets

Venous blood (2 mL) was collected, and the peripheral blood lymphocyte subsets were first labeled with fluorescently labeled monoclonal antibodies. B cells (CD19^+^), total T cells (CD3^+^), CD8^+^ T cells (CD3^+^CD8^+^), CD4^+^ T cells (CD3^+^CD4^+^), and natural killer cells (CD16^+^CD56^+^). Cells were stained in 6-color TBNK Reagent (BD Multitest) that included CD3-FITC, CD16/CD56-PE, CD45-PerCP-Cy5.5, CD4-PE-Cy7, CD19-APC, and CD8-APC-Cy7. Flow cytometry (BD Biosciences, San Jose, CA, USA) was then performed to complete the lymphocyte subpopulation analysis.

### Clinical microbiology testing

#### Microbiological culture

BALF was inoculated in blood agar, chocolate agar, and MacConkey agar plates; placed in a 5% to 10% carbon dioxide environment; and incubated at 35°C for 24 to 48 hours. Fungal culture required two temperatures of 28°C and 35°C with a 5- to 7-day incubation period, during which bacterial or fungal growth on the plate was observed daily. Initial determination of specific microorganisms was performed based on the different colony morphologies and staining on the medium.

#### Mass spectrometry

Suspicious colonies on the medium were selected for further identification of bacterial or fungal taxa by matrix-assisted laser desorption/ionization time-of-flight mass spectrometry.

#### Drug sensitivity testing

For suspected growth of *Streptococcus pneumoniae*, suspicious colonies on the blood agar plate were selected for further purification. The optochin test was performed for identification and drug sensitivity testing of microorganisms such as *Escherichia coli* strains *ATCC25922*, *ATCC35218*, *ATCC25923*, *ATCC27853*, *ATCC29213*, *ATCC29212*, *ATCC49619*, *ATCC66027*, and *ATCC49766*. The drug sensitivity test was performed at Shanghai Clinical Laboratory Centre.

#### Detection of common respiratory viruses

Common respiratory viruses such as respiratory syncytial virus, adenoviruses, influenza A and B, parainfluenza virus, and human metapneumovirus were detected using an immunofluorescence test kit (Diagnostic Hybrids, Athens, OH, USA). The procedure is detailed in the **Supplementary Appendix**.

### mNGS

We performed metagenomic sequencing with reference to our previously published studies (Wang et al., 2019; Xu et al., 2020; Wang et al., 2021; Yan et al., 2021; Zhou et al., 2016; Zhou et al., 2019), as described in the **Supplementary Appendix**.

### Metagenomics analysis

#### Microbial ecological diversity

The sequencing data from each sample were first normalized to obtain the number of sequenced reads of individual microorganisms out of 1 million total sequenced reads [i.e., reads per million (rpm)]. The microbial ecological diversity index of the sample was then calculated based on the rpm value of each microorganism in the sample, including the abundance (i.e. the sum of rpm of all microorganisms), number of species, and alpha diversity index (Chao1 diversity index and Shannon diversity index). The Chao1 diversity index and Shannon diversity index were calculated using the vegan package of R software (version 3.6.1).

#### Determination of pathogenic microorganisms

We referred to a previously published article (Yan et al., 2021) for the determination of pathogenic microorganisms in BALF, as described in the **Supplementary Appendix**.

### Correlation/regression analysis

Pearson correlation coefficients and Spearman correlation coefficients for each microbial ecological diversity index and clinical phenotype were first calculated by the cor.test() function of R software (version 3.6.5). Next, the lm() function of R software (version 3.6.5) was used to perform a linear fit of the microbial ecological diversity index to the clinical phenotypes, was then the ggplot2 package of R software was used to draw the scatterplot. Statistically significant p-values were obtained by the Wilcoxon rank sum test function (wilcox.test) of R software.

## Results

### Clinical information

This study involved 126 children in the PICU, including 84 with community-acquired pneumonia (CAP) and 42 with hospital-acquired pneumonia (HAP). Their mean maximum body temperature was 39°C ± 0.95°C, mean heart rate was 160 ± 19 beats per minute, mean total duration of hospitalization was 37 ± 29 days, and mean number of days in the PICU was 30 ± 29 days. The 30-day mortality analysis revealed 24 deaths and 16 cases of abandoned treatment, accounting for 29.4% of all patients. In total, 86.5% (109/126) of patients had combined respiratory failure, 24.6% (31/126) had combined severe pneumonia, 24.6% (31/126) had combined sepsis, 5.5% (7/126) had septic shock, one had encephalitis, and four were being hospitalized after cord blood stem cell transplantation.

The mean serum CRP concentration was 67 ± 56 mg/L, PCT concentration was 8.9 ± 19 ng/mL, LPS concentration was 1.0 ± 7.6 pg/mL, and IL-6 concentration was 390 ± 850 pg/mL. The white blood cell (WBC) count was 18 ± 12 × 10^9^ cells/L, CD3^+^ lymphocyte count was 1.2 ± 1.3 × 10^9^ cells/L, CD4^+^ T cell count was 6.8 ± 8.7 × 10^8^ cells/L, CD8^+^ T cell count was 5.0 ± 5.9 × 10^8^ cells/L, and NK cell count was 1.4 ± 1.8 × 10^8^ cells/L. Pathogenic microorganisms were detected in 81 patients based on conventional clinical microbiological testing methods such as blood and other cultures.

### Use of mNGS of BALF samples to identify pathogenic microorganisms associated with clinical symptoms

**Figure 1:**
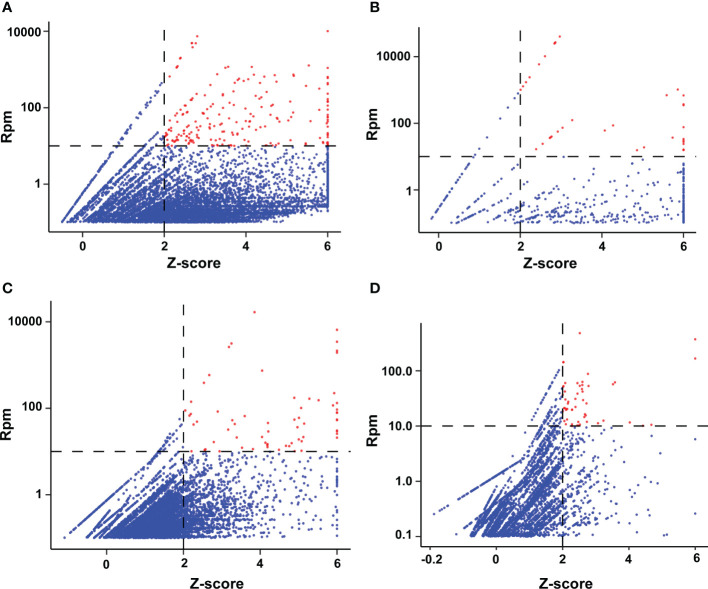
Distribution of microbial abundance and z-score detected by mNGS of BALF samples. **(A)** Bacteria; **(B)** Viruses; **(C)** Fungi; **(D)** Protozoa. An rpm value of <0.01 abundance defaults to a log10 (rpm) of −2 for that microorganism in that sample, and a z-score pf >6 defaults to 6 for all samples. The red dots represent potential pathogens in our selected samples.

mNGS was performed on 126 samples [at the DNA level in most (101/126, 80.1%) and at the RNA level in a smaller number (25/126, 19.8%)]. Each sample sequenced yielded 2.6 ± 1.4 × 10^7^ clean sequences. The potentially pathogenic microorganisms in the samples were further identified by considering both the relative abundance of the microorganisms in the samples to be tested and the outlier levels (z-score) in all samples, following the method used in our previous study (Yan et al., 2021). The relative abundance and z-score distribution of the pathogenic microorganisms obtained are shown in **Figure 1**.

**Figure 2:**
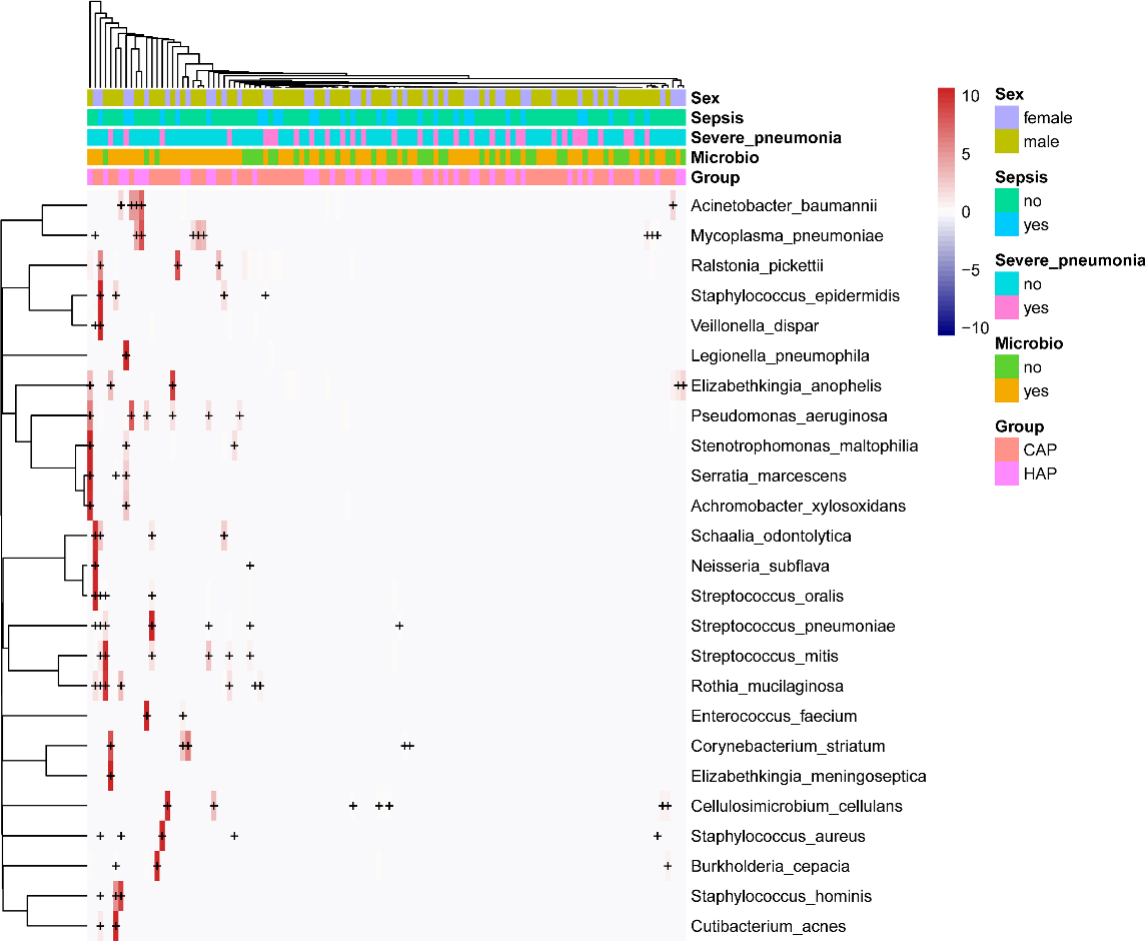
Heat map of potentially pathogenic bacteria detected by mNGS of BALF samples. For the top 25 bacteria, each horizontal row represents a sample, and each vertical column represents potentially pathogenic bacteria. The plus and minus signs in the graph represent significant positive and negative correlations, respectively. The clinical phenotype is at the top, and the color block on the right represents the value of the specific clinical phenotype.

Nucleic acid sequences of 88 pathogenic bacteria were detected, including 9 species of *Streptococcus* (*Streptococcus agalactiae*, *Streptococcus australis*, *Streptococcus milleri*, *Streptococcus mitis*, *Streptococcus oralis*, *Streptococcus parasanguinis*, *Streptococcus pneumoniae*, *Streptococcus pseudopneumoniae*, and *Streptococcus salivarius*), 7 species of *Staphylococcus* (*Staphylococcus aureus*, *Staphylococcus capitis*, *Staphylococcus epidermidis*, *Staphylococcus haemolyticus*, *Staphylococcus hominis*, *Staphylococcus saprophyticus*, and *Staphylococcus warneri*), and 3 species of *Acinetobacter* (*Acinetobacter baumannii*, *Acinetobacter johnsonii*, and *Acinetobacter nosocomialis*). The top 25 bacteria are listed in **Figure 2**. In total, 13 viral nucleic acid sequences were detected, including 4 human herpesviruses [*Human alphaherpesvirus 1*, *Human betaherpesvirus 5* (also known as human cytomegalovirus), *Human betaherpesvirus 6B*, and *Human gammaherpesvirus 4* (also known as Epstein–Barr virus)], *Human mastadenovirus B*, and *Human polyomavirus 4*. All viruses are shown in **Supplementary Figure 1**. Nucleic acid sequences were detected for 39 pathogenic fungi, including 4 *Aspergillus* species (*Aspergillus fischeri*, *Aspergillus fumigatus*, *Aspergillus mulundensis*, and *Aspergillus nidulans*) and *Pneumocystis jirovecii*; the other fungi are shown in **Supplementary Figure 2**. Nucleic acid sequences were detected for 27 protozoans, including 10 protozoa of the genus *Plasmodium* (*Plasmodium falciparum*, *Plasmodium gaboni*, *Plasmodium gallinaceum*, *Plasmodium gonderi*, *Plasmodium knowlesi*, *Plasmodium malariae*, *Plasmodium reichenowi*, *Plasmodium relictum*, *Plasmodium sp*, and *Plasmodium yoelii*) and 2 protozoa of the genus *Trypanosoma* (*Trypanosoma cruzi* and *Trypanosoma theileri*). The others are specified in **Supplementary Figure 3**.

### Correlation of pathogenic microbial abundance in BALF with serum inflammatory indicators

**Figure 3:**
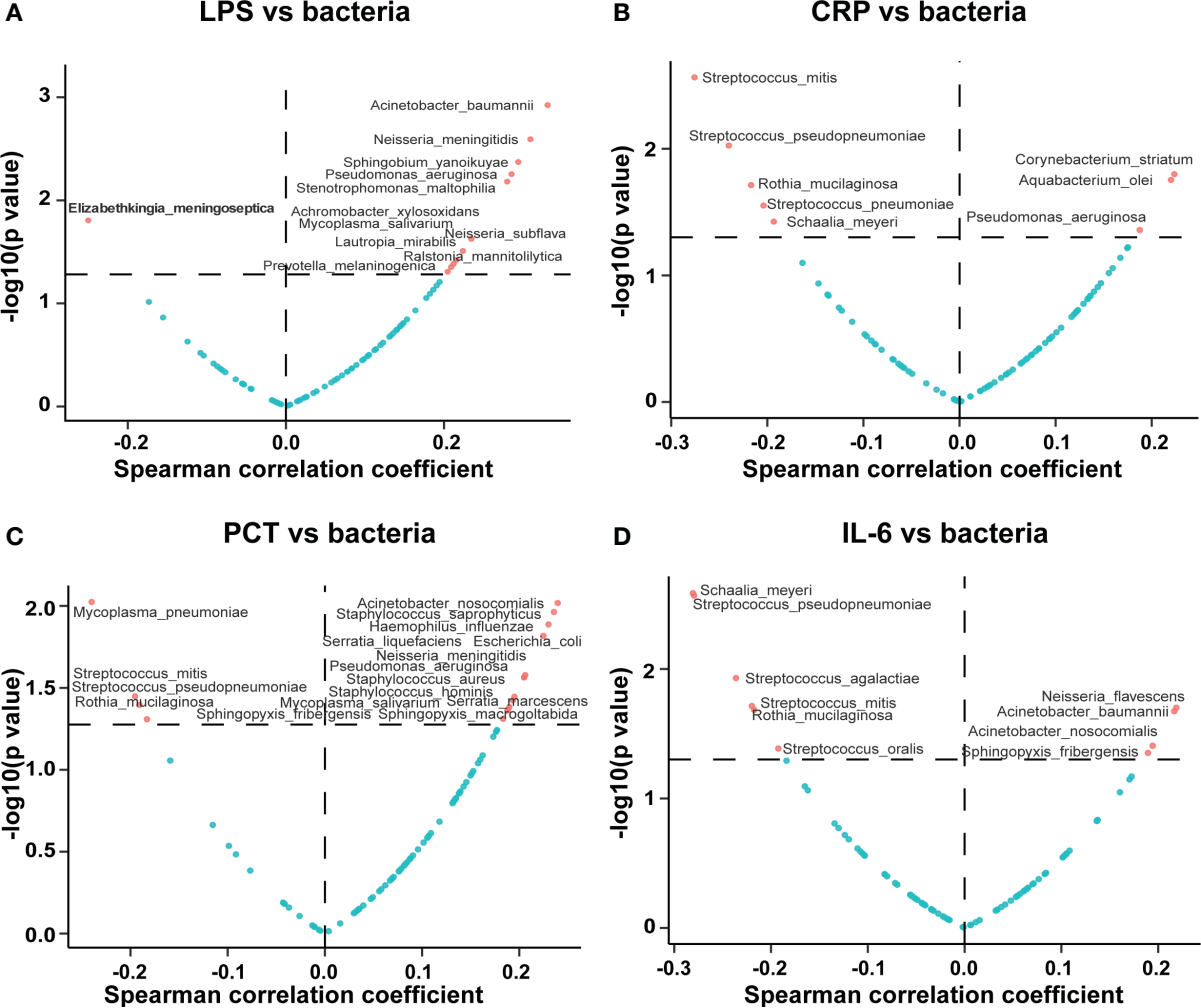
Correlations of potentially pathogenic bacteria in BALF with serum inflammatory indicators. The volcano plot displays the correlations of the potentially pathogenic bacteria in BALF with the serum **(A)** LPS, **(B)** CRP, **(C)** PCT, and **(D)** IL-6 concentrations. The horizontal coordinate represents the Spearman correlation coefficient, and the vertical coordinate represents the negative logarithm of the p-value of the correlation between the clinical phenotype and potential pathogen; i.e., −log10 (p value). Each point in the graph represents a potentially pathogenic microorganism, and those screened for significant differences have been marked in red.

We found that the serum LPS concentration was correlated with the pathogenic bacterial abundance in BALF. Notably, most of the 12 serum LPS-associated bacteria identified were positively correlated with the serum LPS concentration, including *Acinetobacter baumannii*, *Neisseria meningitidis*, *Neisseria subflava*, *Pseudomonas aeruginosa*, *Stenotrophomonas maltophilia*, *Mycoplasma salivarium*, and several others; the full results are shown in **Figure 3A**. We also found that the serum CRP concentration was correlated with the pathogenic bacterial abundance in BALF, with *Pseudomonas aeruginosa* and *Corynebacterium striatum* positively correlated with the serum CRP concentration, as shown in **Figure 3B**. Moreover, the serum PCT concentration was correlated with the abundance of pathogenic bacteria in BALF; the abundance of *Staphylococcus aureus*, *Staphylococcus hominis*, *Staphylococcus saprophyticus*, *Haemophilus influenzae*, *Pseudomonas aeruginosa*, and *Mycoplasma salivarium* was positively correlated with the serum PCT concentrations, as shown in **Figure 3C**. Likewise, we found that serum IL-6 concentration was correlated with the abundance of pathogenic bacteria in BALF, with positive correlations noted for *Acinetobacter baumannii* and *Acinetobacter nosocomialis*. Notably, we found that certain *Streptococcus* spp. Pathogenic bacteria in BALF, such as *Streptococcus pseudopneumoniae*, *Streptococcus agalactiae*, *Streptococcus oralis*, and *Streptococcus mitis*, were negatively correlated with the serum IL-6 concentration, as shown in **Figure 3D**.

### Correlation of pathogenic microbial abundance in BALF with total WBC count and T-lymphocyte count

The data showed that the total WBC count was negatively correlated with the abundance of pathogenic bacteria in BALF, such as *Pseudomonas aeruginosa*, *Prevotella jejuni*, *Streptococcus australis*, *Achromobacter xylosoxidans*, *Sphingopyxis fribergensis*, *Actinomyces pacaensis*, *Actinomyces naeslundii*, *Herbaspirillum huttiense*, and others (**Supplementary Figure 4A**). We also found that the total WBC count was negatively correlated with the abundance of *Human betaherpesvirus 6B* and *Human gammaherpesvirus 4* in BALF (**Supplementary Figure 4B**) and with the abundance of fungi in BALF such as *Pneumocystis jirovecii* and *Aspergillus terreus* (**Supplementary Figure 4C**).

Total T cell count was correlated with the abundance of pathogenic bacteria in BALF such as *Staphylococcus aureus*, *Staphylococcus haemolyticus*, and *Staphylococcus warneri* (**Supplementary Figure 5A**). Notably, we found that the abundance of *Staphylococcus aureus, Staphylococcus haemolyticus*, and *Staphylococcus warneri* in BALF was negatively correlated with the counts of blood CD4^+^ T cell, CD8^+^ T cell, and NK cell (**Supplementary Figures 5B–D**).

### Differences in microbiological composition of BALF between patients with CAP and HAP

**Figure 4:**
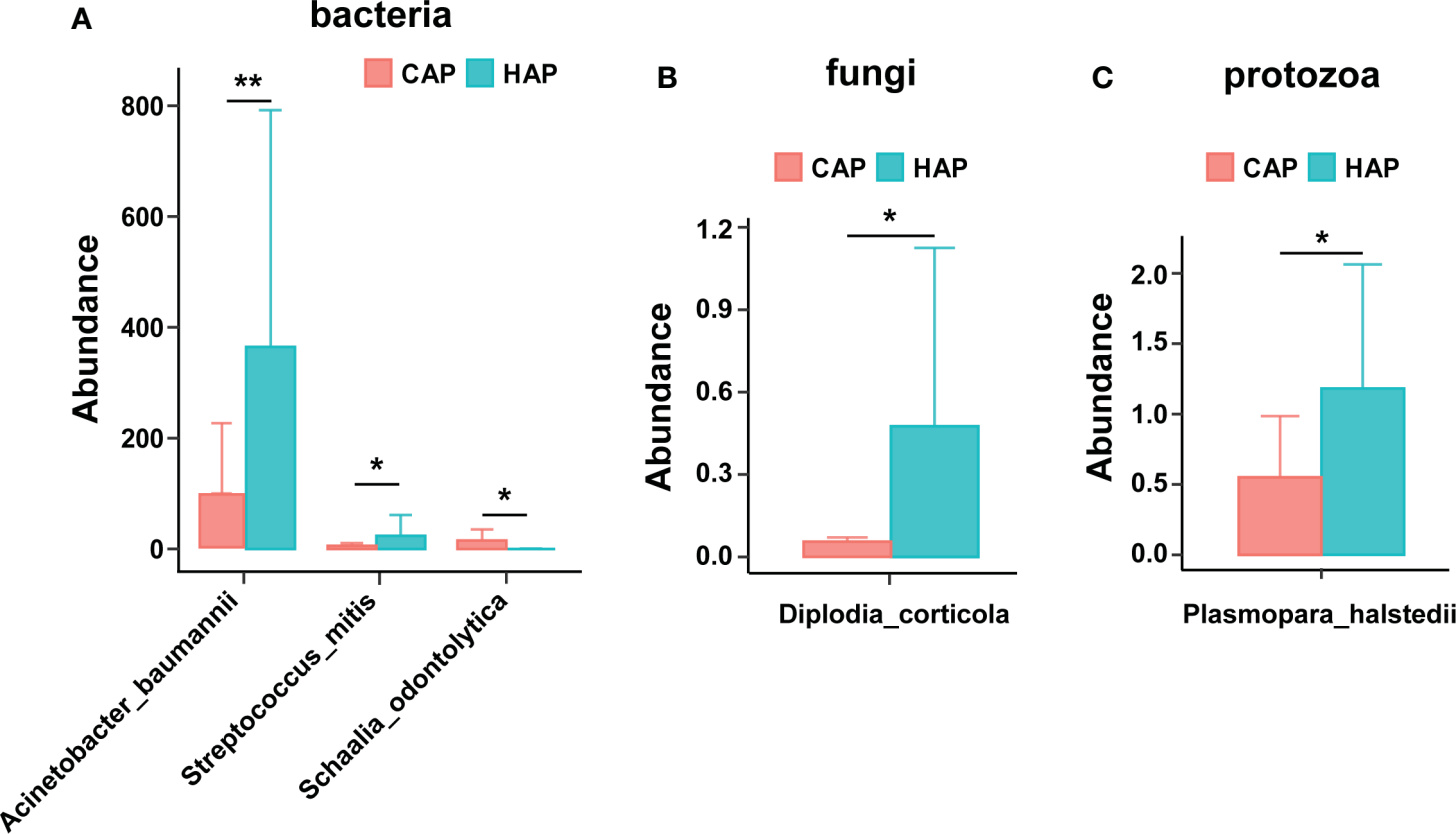
Significant differences in pathogenic microbial composition of bronchoalveolar lavage fluid in the CAP and HAP groups. Significant differences were noted in potentially pathogenic **(A)** bacteria, **(B)** fungi, and **(C)** protozoans between patients with CAP versus HAP. *p < 0.05, **p < 0.01.

We performed a differential analysis of the pathogenic microbial composition of BALF in patients with CAP and HAP and found 3 pathogenic bacteria that were present at significantly different levels. There were significantly greater amounts of *Acinetobacter baumannii* and *Streptococcus miti* in patients with HAP than that in patients with CAP, while the amount of *Schaalia_odontolytica* was significantly lower in the patients with HAP than that in the patients with CAP. We also found a significantly higher amount of the potentially pathogenic fungus *Diplodia corticola* and the potentially pathogenic parasite *Plasmopara halstedii* in the BALF of patients with HAP than of patients with CAP (**Figure 4**).

### Correlation of mortality and oxygenation index of severe pneumonia with pathogenic microbial composition of BALF

**Figure 5:**
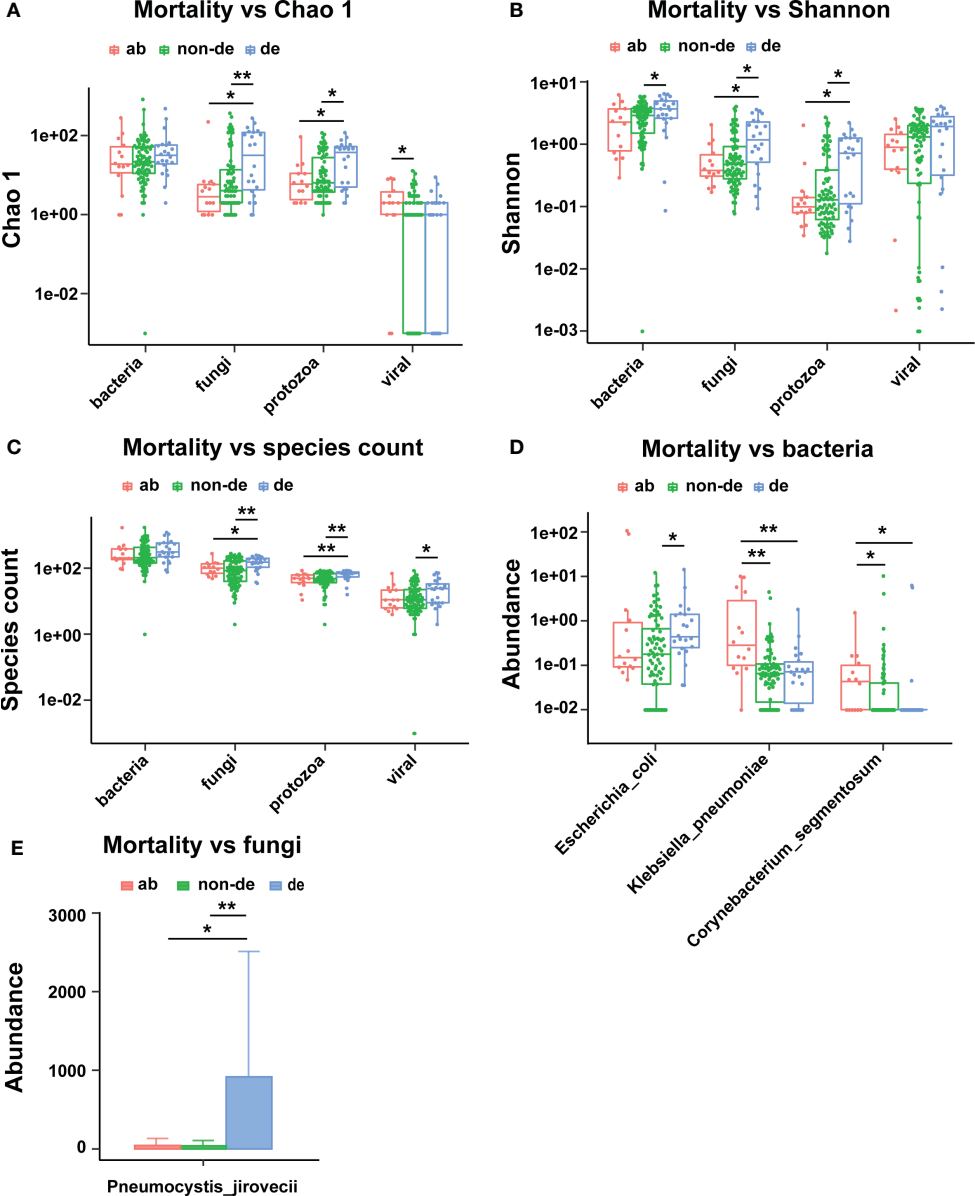
Ecological diversity and composition of pathogenic microorganisms in BALF associated with mortality. **(A)** Mortality associated with Chao1; **(B)** Mortality associated with Shannon index; **(C)** Mortality associated with species count; **(D)** Mortality associated bacteria; **(E)** Mortality associated with fungi. ab, abandoned; de, deceased; non-de, non deceased. *p < 0.05, **p < 0.01.

We assessed the correlation between lethal phenotypes (death or abandonment of treatment) and the ecological diversity of pathogenic microorganisms in BALF. The results showed that the fungal and protozoa richness (Chao1 index) was significantly higher in BALF from deceased than non-deceased patients (**Figure 5A**); the fungal and protozoal diversity (Shannon index) was also significantly higher in BALF from deceased than non-deceased patients (**Figures 5B, C**). We also assessed the correlation between lethal phenotypes (death or abandonment of treatment) and the pathogenic microbial composition of the BALF. The results showed that *Escherichia coli* abundance was significantly higher in the BALF from deceased than non-deceased patients. Notably, we found significantly higher abundance of *Klebsiella pneumoniae* and *Corynebacterium segmentosum* in the BALF of patients in the abandoned treatment group than in the deceased/non-deceased group (**Figure 5D**). Additionally, there was a significantly higher abundance of *Pneumocystis jirovecii* in the BALF of deceased patients than in both the non-deceased and abandoned treatment groups (**Figure 5E**).

**Figure 6:**
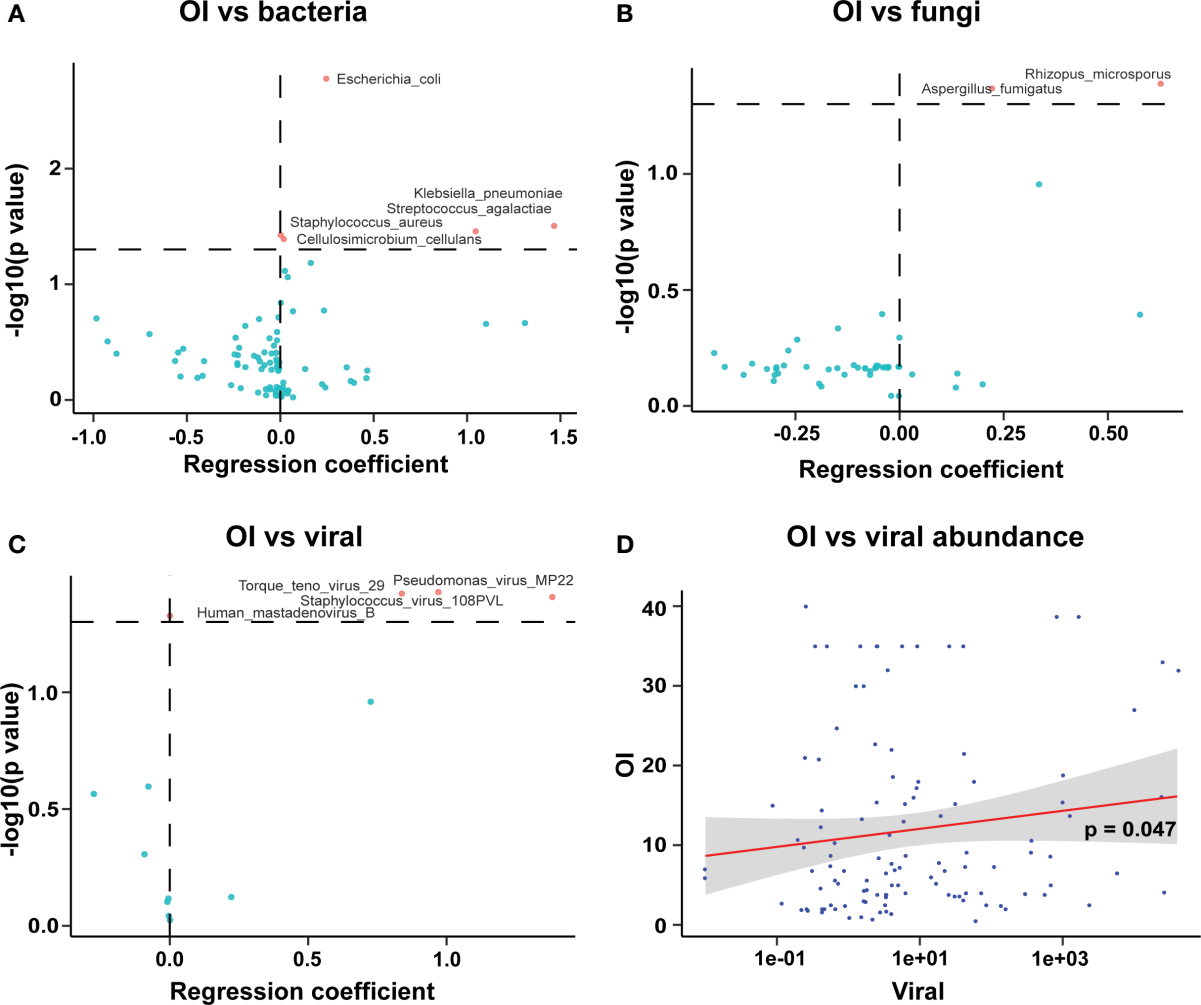
Correlation of oxygenation index with pathogenic microorganisms in BALF of patients with severe pneumonia. **(A)** The OI was positively correlated with the abundance of pathogenic bacteria in BALF; **(B)** The OI was positively correlated with the abundance of pathogenic fungi in BALF; **(C)** The OI was positively correlated with the abundance of viruses in BALF; **(D)** The OI was correlated with the overall abundance of viruses (viral abundance) in BALF.

OI reflects the severity of disease in patients with severe pneumonia. The data showed that OI was associated with increased abundance of pathogenic bacteria in BALF, including *Escherichia coli*, *Klebsiella pneumoniae*, *Streptococcus agalactiae*, and *Staphylococcus aureus* (**Figure 6A**). Moreover, The OI was positively correlated with an increased abundance of the fungi *Aspergillus fumigatus* and *Rhizopus microspores* in BALF (**Figure 6B**) and with an increased abundance of the viruses *Human mastadenovirus B* and *Torque teno virus 29* in BALF (**Figure 6C**). In addition, there was a weak positive correlation between OI and the overall viral abundance (**Figure 6D**).

### Sepsis and immunodeficiency in patients with severe pneumonia associated with BALF microorganisms

Among patients with severe pneumonia, the BALF fungal and parasite species richness (Chao1 index), Shannon diversity, and number of species were significantly higher in patients with than without sepsis (**Supplementary Figure 6**). Additionally, the total number of bacterial species in BALF was significantly higher in patients with than without immunodeficiency (**Supplementary Figure 7A**). Moreover, the abundance of *Elizabethkingia anophelis* in BALF was significantly higher in patients with than without immunodeficiency (**Supplementary Figure 7B**). Notably, we found that the abundance of *Human betaherpesvirus 5* (i.e., human cytomegalovirus) in BALF was significantly higher in patients with than without immunodeficiency (**Supplementary Figure 7C**).

## Discussion

In this retrospective study, we included 126 BALF samples from children with severe pneumonia admitted to the PICU with the aim of exploring the potential microbiome composition. As for mNGS-based pathogen identification and aiding clinical decision-making, this is a challenging task. In this study, although all BALF samples were completed with mNGS testing, the turnaround time for performing mNGS testing from early 2018 to early 2020 was several days or even a week, with most samples being taken at 48-72 hours. Also, considering that the condition of PICU patients is rapidly changing; So we did not wait for mNGS results to start clinical decision-making. Instead, we refer to the results of conventional clinical immunological and microbiological testing methods such as lymphocyte subtypes, inflammatory factor markers, and pathogenic microbial cultures for initial determination of pathogenic infection and clinical decision-making. Happily, it is now possible to obtain mNGS results in less than 24 hours and to guide clinical decision making based on mNGS in our hospital.

This aim of the present study is to resolve the microbiome composition of BALF from children with severe pneumonia in PICU by mNGS technique and to identify some microbiome features associated with clinical indicators. The mNGS performed on BALF samples facilitates the rapid identification of pathogens of critical illnesses. However, as with traditional interpretation of metagenomic data, the interpretation of mNGS results is challenging. To gain a more comprehensive understanding of the microbiome composition of BALF, we performed a comprehensive assessment of microbial composition in BALF according to four pathogenic microbial communities: bacterial, viral, fungal, and protozoan, with reference to traditional metagenomic data analysis methods. By correlating microbial ecological diversity indicators, high abundance of pathogenic microorganisms with conventional clinical indicators such as lymphocyte subtypes, inflammatory markers, etc., some indicative microecological indicators or pathogenic microorganisms associated with disease were identified.

### Correlation of abundance of potentially pathogenic bacteria in BALF with serum inflammatory markers and lymphocyte subtypes

In the present study, we found that the BALF bacterial diversity indices, including the Chao1 diversity index and overall abundance, were positively correlated with the serum LPS concentration; the bacterial species count was positively correlated with the serum PCT concentration; and that the *Pseudomonas aeruginosa* abundance in BALF was positively correlated with the serum LPS, CRP, and PCT concentrations and negatively correlated with the whole blood WBC count and CD4^+^ T cell count.

*Pseudomonas aeruginosa* is a conditionally pathogenic bacterium widely present in nature, human skin, intestine, and respiratory tract; studies have shown that P. aeruginosa is an important causative agent of hospital-acquired pneumonia (HAP) and ventilator-associated pneumonia (VAP) (Pang et al., 2019); notably, *P. aeruginosa* is prone to cause intranasal and ventilator-associated pneumonia in immunocompromised and hospitalized patients (Killough et al., 2022). Consistent with this, we found that *P. aeruginosa* abundance in BALF was negatively correlated with whole blood WBC counts and CD4^+^ T cell counts; also, in this study, we found that the number of bacterial species detected in BALF was elevated in the immunodeficient group relative to the non-immunodeficient group (**Supplementary Figure 7A**). In short, this implies that the rise in the abundance of *P. aeruginosa* may be associated with immunocompromised or impaired immunity in patients. *Pseudomonas aeruginosa* is the most common multidrug-resistant Gram-negative bacterium and a common cause of ventilator-associated pneumonia in patients in ICUs. Ventilator-associated pneumonia caused by *Pseudomonas aeruginosa* infection is characterized by high morbidity and mortality (Zhuo et al., 2008; Valencia and Torres, 2009). Experimental models suggest that ventilator-induced lung injury is associated with increased bronchial vascular permeability in BALF, higher cell counts and protein concentrations, and increased infiltration of inflammatory cells into lung tissue (Wolters et al., 2009). Cytokines are small proteins that communicate through intercellular signaling and can be considered immunomodulators of immune and inflammatory responses (Spelman et al., 2006). Human studies have shown that cytokine/chemokine release and leukocyte recruitment contribute to ventilator-associated lung injury (Halbertsma et al., 2005) and that IL-6, an inflammatory marker of ventilator-associated lung injury, may contribute to alveolar barrier dysfunction in patients with acute respiratory distress syndrome (Wolters et al., 2009). IL-6 is an important pro-inflammatory cytokine that is released during infection or tissue injury; IL-6 is mainly expressed by natural immune cells such as monocyte macrophages, but can also be produced by Th2 cells, vascular endothelial cells, and fibroblasts (Kang and Kishimoto, 2021). In the present study, we observed a correlation between serum IL-6 levels and the abundance of specific pathogenic microorganisms in BALF (**Figure 3D**). Tsay et al. found that, like mechanical ventilation, intranasal perfusion with *P. aeruginosa* also induced IL-6 expression in the lungs (Tsay et al., 2016). We did not find a correlation between *P. aeruginosa* abundance and serum IL-6 levels in BALF and could not identify the source of serum IL-6.

*Acinetobacter baumannii*, a Gram-negative aerobic bacterium, is listed by the World Health Organization as posing the greatest threat to human health and as being in urgent need of new antibiotics (World health organization, 2017; Lee et al., 2017). In recent years, isolates of *Acinetobacter baumannii* have been recovered from a variety of extra-hospital sources (e.g., vegetables, water treatment plants, and fish and shrimp farms) in addition to known natural habitats (soil and moist environments), and the wide range of sources of this bacterium might explain the occurrence of community-acquired infections (Eveillard et al., 2013). *Acinetobacter baumannii* is a serious pathogen involved in hospital-acquired and community-acquired infections (Dijkshoorn et al., 2007). Cases of CAP caused by *Acinetobacter baumannii* are rare in Europe and the United States (Serota et al., 2018); instead, most cases of CAP caused by *Acinetobacter baumannii* occur in countries with tropical or subtropical climates. *Acinetobacter baumannii* is an emerging pathogen in the Asia-Pacific region, with a high prevalence in Hong Kong, Singapore, Taiwan, Korea, and Australia (Kim et al., 2018). In the present study, we found that the abundance of *Acinetobacter baumannii* in BALF of children in the PICU was positively correlated with the serum IL-6 and LPS concentrations. Notably, the abundance of *Acinetobacter baumannii* in BALF was significantly higher in patients with HAP than CAP; this may be related to the fact that *Acinetobacter baumannii* is more common in hospitals than in the community. The primary target of *Acinetobacter baumannii* is patients in ICUs, and the use of antibiotics has led to outbreaks and epidemics of infections caused by multidrug-resistant *Acinetobacter baumannii* in hospitals (Dijkshoorn et al., 2007; Ramirez et al., 2020).

### Correlation of potentially pathogenic viral infections in BALF of children in PICU with immunodeficiency and pneumonia severity

Respiratory viral infections are an important cause of pneumonia in children. Viral bronchiolitis is the most common cause of respiratory failure in young children requiring invasive ventilation, and coinfections such as bacterial and fungal infections may prolong and complicate hospital stays in the PICU (Wiegers et al., 2019; Li et al., 2020b). We performed mNGS on BALF from children in the PICU and found that viral nucleic acid sequences were present; in addition, the Chao1 diversity index of viruses was positively correlated with the maximum body temperature on admission and negatively correlated with the CD3^+^ lymphocyte count. As a microbial ecology index, the viral Shannon diversity index reflects both the number of viruses and their abundance. In calculating the Shannon diversity index of BALF samples, the included viruses contain both eukaryotic viruses and prokaryotic viruses such as phages, and usually the number of phage species is more abundant than eukaryotic viruses. We observed that the Shannon diversity index of viruses was negatively correlated with the counts of tatol T cell, CD4^+^ T cell, CD8^+^ T cell and NK cell, suggesting whether certain viruses may have a protective role, such as phages. Considering that phages usually act as commensal microorganisms and not as pathogenic microorganisms; therefore, they have not been analyzed and discussed here.

The number of ICU admissions for viral infections is rising. The Herpesviridae family, including *Human gammaherpesvirus 4* (i.e., Epstein–Barr virus) and *Cytomegalovirus*, can be reactivated in ICU patients, and such viral activation is associated with morbidity and mortality (Cantan et al., 2019). Consistent with this, we found that the abundance of *Human betaherpesvirus 6B* and *Human gammaherpesvirus 4* was negatively correlated with the total WBC count. Additionally, the abundance of *Human betaherpesvirus 5* (i.e., human cytomegalovirus) in the BALF of patients with severe pneumonia was significantly higher in those with than without immunodeficiency, suggesting a possible correlation between immune system dysregulation and viral activation. We also found that the severity of pneumonia (i.e., the OI) was positively correlated with the overall viral abundance in the BALF; notably, we found that the OI was positively correlated with an increase in the abundance of the viruses *Human mastadenovirus B* and *Torque teno virus 29* in BALF. In summary, these findings suggest a correlation between the viral load in BALF and the severity of pneumonia.

### Detection of potentially pathogenic eukaryotes in BALF of children with severe pneumonia in the PICU and their association with death and sepsis

Fungi are another important cause of respiratory infections (Schmidt et al., 2018). We found significantly higher species richness (Chao1 index), Shannon diversity, and number of species of pathogenic fungi in the BALF of patients with than without sepsis in the present study. *Aspergillus*, *Pneumocystis*, and *Cryptococcus* are the key fungal pathogens known to cause respiratory infections (Hayes and Denning, 2013). *Pneumocystis jirovecii* causes pneumocystis pneumonia, a life-threatening disease in the ICU with a high mortality rate, particularly in immunocompromised patients (Salzer et al., 2018; Schmidt et al., 2018). Schmidt et al. (Schmidt et al., 2018) found that the overall hospital mortality rate was 25.4% and increased to 58.0% if ICU admission was required. In line with this, we found a significantly higher abundance of *Pneumocystis jirovecii* in the BALF of deceased patients than in both non-deceased patients and patients for whom treatment was abandoned.

Patients in the ICU are more likely to have a history of coinfection with bacteria and fungi than are patients in the general ward (Schauwvlieghe et al., 2018). Beumer et al. (Beumer et al., 2019) found that the incidence of coinfection with bacteria and fungi in the lungs of patients with influenza was 55.6% among ICU inpatients, which was significantly higher than in patients in the normal ward (20.1%), and *Aspergillus fumigatus* and *Pneumocystis jirovecii* were the predominant coinfecting fungi (Beumer et al., 2019). In line with this, we found that an increase in the abundance of the fungal pathogen *Aspergillus fumigatus* in BALF was positively correlated with the severity (i.e., the OI) of patients with severe pneumonia, while an increase in the abundance of the bacterial pathogens *Klebsiella pneumoniae*, *Streptococcus agalactiae*, and *Staphylococcus aureus* in BALF of children in the PICU was also positively correlated with the severity (i.e., the OI) of patients with severe pneumonia.

Protozoan coinfection increases the risk of pneumonia in children (D'Acremont et al., 2014; Edwards et al., 2015). We found significantly higher protozoan species richness (Chao1 index), Shannon diversity, and number of species in the BALF of patients with than without sepsis in the present study; we also detected nucleic acid sequences of *Plasmodium* and *Trypanosoma* in the BALF of children in the PICU. Notably, protozoa have a large genome, and the mNGS results only cover a very small part of their genome. Although in-depth validation is needed, our results suggest the possibility of protozoan coinfection.

This study aimed to gain a comprehensive understanding of the microbiome composition in the BALF of children with severe pneumonia in the PICU. Although uncommon, protozoa such as parasites may also represent the “potential” pathogenic microbial composition in BALF (Van den Steen et al., 2010; Deroost et al., 2013; Wihokhoen et al., 2016; Kumar et al., 2022). Therefore, after metagenomic sequencing, the sequenced reads were aligned to bacterial, viral, and fungal databases, as well as to protozoan databases, and the “potential” protozoan composition was obtained. Considering that the protozoan genome size is larger than that of bacteria, viruses, and fungi, it is difficult to determine whether protozoa actually exist with a limited number of reads, but we just try to understand that the nucleic acid sequence of the parasite was detected. In the next step, we need to verify the existence of protozoa by 18S rRNA sequencing technology.

Considering that specific pathogenic microorganisms were detected only in a small number of BALF samples; therefore, we did not perform a correlation/regression analysis of the abundance of specific pathogenic microorganisms and clinical indicators, or a multiple linear regression analysis. Instead, we performed a logistic linear regression of the microecological diversity indicators with the clinical indicators, and the results are shown in **Figure 6D**, where it can be seen that OI was positively correlated with the abundance of pathogenic bacteria in BALF. Indeed, the correlation is weaker, but reflects a trend.

All patients included in this study were from pediatric intensive care units; usually, DNA viruses, bacteria, fungi, and mycoplasma are the predominant pathogenic microorganisms associated with the respiratory tract in children, with RNA viruses accounting for a relatively small proportion. The mNGS used in this study cannot take into account both DNA and RNA viruses. According to the clinical empirical judgment of two specialized physicians, 25 of 126 patients were inclined to RNA virus infection, in order to obtain the test results in the shortest time and save clinical costs. We only tested the above 25 cases at the RNA level and 121 cases at the DNA level, and the test results were also consistent with the empirical judgment results. Of course, the mNGS technique in this study cannot take into account both DNA and RNA viruses, and there may be missed detection. Nowadays, our team has developed the mNGS pathogen capture technique based on mNGS, and the development of this technique will make up for this deficiency and provide a new direction of choice for pathogenic diagnosis in line with clinical thinking.

This study had some limitations. The inflammatory marker testing using BALF samples can provide direct insight into the impact of different pathogenic infections on pneumonia outcomes. However, since the volume of BALF obtained from children is limited; therefore, BALF samples were not used for inflammatory marker assays in addition to pathogenic assays; as an alternative strategy, we performed inflammatory marker assays on blood samples. The pathogenic nucleic acid sequences we identified in BALF in the PICU require in-depth validation, including completion by clinical microbiological methods such as BALF culture and antibody testing. Further, multicenter scale-up validation is also needed for an in-depth assessment of mNGS results relevance to clinical symptoms in children with severe pneumonia in the PICU.

## Conclusions

mNGS of BALF from children with severe pneumonia in the PICU revealed potentially pathogenic bacterial infections. The increased bacterial Chao1 diversity index and overall abundance in the BALF were positively correlated with serum inflammatory indicators, and altered abundance of potentially pathogenic bacteria was correlated with serum inflammatory indicators and altered lymphocyte subtypes. In addition, there was the potential for coinfection with viruses in these children as shown by the correlation of the abundance of potentially pathogenic viruses in BALF with the presence of immunodeficiency and the severity of pneumonia. We also found the potential for fungal coinfections in these children as indicated by the positive association of increased diversity of potentially pathogenic fungi in BALF with the occurrence of death and sepsis.

## Data availability statement

The raw data that support the findings of this study have been deposited into CNGB Sequence Archive (CNSA) of China National GeneBank DataBase (CNGBdb) with accession number CNP0004045.

## Ethics statement

The studies involving human participants were reviewed and approved by The Research Ethics Board of the Children’s Hospital of Fudan University (IRB protocol number: 2019-312). Written informed consent to participate in this study was provided by the participants’ legal guardian/next of kin. Written informed consent was obtained from the individual(s), and minor(s)’ legal guardian/next of kin, for the publication of any potentially identifiable images or data included in this article.

## Author contributions

CZ drafted the initial version of manuscript; TL performed investigation, formal analysis, and visualization; YW, WC, JL, and JT were responsible for resources and data curation; ZZZ, XZ and ZYZ validated the data; MM and MW performed Writing - Review & Editing; MW performed visualization and software; GY and GL conceived and supervised the study; All authors read and approved the final draft for publication.
