# Supplementary material for: Metagenomic next-generation sequencing of bronchoalveolar lavage fluid from children with severe pneumonia in pediatric intensive care unit
Source: Front Cell Infect Microbiol. 2023 Mar 16;13:1082925. doi: 10.3389/fcimb.2023.1082925 (PMC10064343; doi:10.3389/fcimb.2023.1082925)
Supplement: Supplementary file 1 [file DataSheet_1.docx]

**Supplementary Appendix**

**Supplementary methods**

***Serum inflammatory markers* LPS assay**. Using a sterile heatless blood collection tube, 2-3ml of venous blood is collected and mixed. The plasma is separated by centrifugation at low speed (400g/min) i.e. 1710rpm/min for 10 minutes. Add 0.1 ml of plasma to 0.9 ml of sample preparation solution, vortex with a vortex mixer and place in a "smart thermostat" (75°C) for 10 minutes, then leave at room temperature or in a cooling zone for 5 minutes. Add 50 μl of reagent master mix to each tube. Add 100μl of the cooled plasma to the main reagent tube, shake gently and insert immediately into the LKM dynamic tube tester in the programmed position for reaction. Click on "Dynamic Curve" to see a real-time curve of the reaction. At the end of the reaction, the content is automatically calculated by the software analysis system. The test results are found in the software analysis system. **Procalcitonin (PCT), Interleukin-6 (IL-6) assay**. 2ml of venous blood was collected from the child using a sterile serum tube, centrifuged at 3000rpm for 5 minutes and PCT and IL-6 were quantified in the serum using a fully automated electrochemiluminescent immunoassay system (Roche Cobas_602, Basel, Switzerland). **C-Reactive Protein (CRP) assay**. 0.5-1ml of venous blood is collected from the child using a sterile heparin anticoagulation tube and the CRP concentration is quantified using a fully automated QuikReadgo C-Reactive Protein analyzer (Orion Diagnostica Oy, Helsinki, Finland).

***Detection of common respiratory viruses*** aspirate the patient's nasopharyngeal secretions into a 2 ml saline tube with a negative pressure aspirator, repeatedly blow with a capillary pipette until the mucus is broken up, discard the supernatant and wash the precipitate 2 times with PBS repeatedly. The precipitated cells were coated on slides, blown dry and fixed in cold acetone at 4°C for 8-10 minutes and air-dried; 5 μL of different viral antibodies were added to each cell spot, placed in a wet box and incubated at 37°C for 30 minutes; the slides were rinsed once in PBS and then placed in a PBS wash tank for 2 full washes of 10 minutes each; excess liquid was wiped off the slides, a drop of sealing solution was added and the slides were sealed with coverslips; fluorescence Microscopic observation (200X, FITC filter) showed fluorescence of apple green in the virus-infected cells; cells not infected with the virus did not fluoresce and were only stained red by Evan's. Two fluorescing cells per cell spot are considered positive. Rhinovirus (RV), Mycoplasma pneumoniae, Chlamydia trachomatis (CT) and assay: Collect nasopharyngeal secretions from the child into a 1ml saline tube, centrifuge at 1000rpm for 3min; aspirate 200μl of supernatant from the specimen. The nucleic acid was extracted using a magnetic bead extraction kit; the nucleic acid was detected using a reverse transcription real-time PCR kit (Daan Gene).

***Metagenomic Next-Generation Sequencing(mNGS).* 1) DNA extraction and pretreatment**: A 0.6-3 ml sample of the patient's alveolar lavage fluid was first inactivated by placing the specimen in a water bath at 65°C for 30 minutes; 600 μL of the alveolar lavage fluid was then placed in a sterile 2.0 mL centrifuge tube, 500 μL of 0.5 mm diameter glass grinding beads were added, sealed with a Parafilm membrane and shaken for 20 minutes at room temperature (2800-3200 rpm) on a vortex shaker. ) to do the wall-breaking process. Then 300 ul of the broken liquid was taken and DNA was extracted using the TIANamp Micro DNA Kit (No. DP316, Tiangen Biochemical Technology, Beijing, China) according to the kit instructions; the final genomic DNA was sonicated to 150-200 bp using a nucleic acid interrupter (Bioruptor Pico, Diagenode, Belgium) and used for library construction. **2) RNA extraction and reverse transcription**: First, 0.5-3 ml of the patient's alveolar lavage fluid is taken and the RNA is extracted using the QIAamp Viral RNA Extraction Kit (QIAampViralRNA Mini Kit, item no. 52904, QIAGEN, Germany), referring to the manufacturer's instructions; then, the RNA is reverse transcribed by Super Script II reverse transcriptase (Thermo Fisher Scientific, USA) to obtain cDNA; The cDNA is further synthesised by DNA polymerase I (item P7050L, Enzymatics, USA) into another complementary DNA; finally, the resulting double-stranded DNA is broken to 150-200 bp fragments by sonication with a nucleic acid interrupter (Bioruptor Pico, Diagenode, Belgium) and used for library construction. **3) Library construction**: First, the end repair, ligation and PCR amplification of the DNA fragments were performed using the MGIEasy Cell-free DNA Library Prep Kit (MGI tech, Shenzhen, China) according to the manufacturer's kit; then, the libraries were quality controlled using the 2100 Bioanalyzer (Agilent, USA) for quality control of the libraries, with a qualified library fragment size of 200-300 bp, and a Qubit dsDNA HS Assay Kit (Thermo Fisher Scientific, USA) for DNA library concentration of >2 ng/μL; finally, the The different samples were mixed in equal amounts of nucleic acid for subsequent operations. **4) High throughput sequencing**: High-throughput sequencing is done by BGISEQ-50 (MGI tech, Shenzhen, China) referring to the manufacturer's instructions, and is briefly described as follows: first, the mixed library with equal nucleic acid amounts is thermally denatured with double-stranded DNA to form single-stranded DNA; then the single-stranded DNA is cyclised to form a single-stranded circular structure and amplified using Rolling circle amplification (RCA) to amplify the single-stranded circular DNA by 2-3 orders of magnitude to obtain DNA Nano Balls (DNB); finally, the DNA is sequenced in a single-ended 50bp pattern. **5) Sequencing data pre-processing**: The sequencing data obtained were first subjected to data quality control to remove low quality and reads with sequence lengths less than 35bp reads to obtain high quality reads; the high quality reads were then compared to the human reference genome (version hg19) by BWA software (version 0.7.15-r1140) to remove contamination from human-derived sequences; the remaining data were based on prinseq software (version 0.20.4) after removing repetitive, low-complexity sequences, and then aligned to the PMDB pathogen database (BGI's internal database of 4945 viruses, 6039 bacteria (excluding Mycobacterium), 174 Mycobacterium, 137 Mycoplasma, Chlamydia 1064 fungi and 234 parasites associated with human diseases); The microbial sequence data obtained after the alignment were annotated according to viruses, bacteria, fungi and parasites.

**Determination of pathogenic microorganisms**: We refer to the method of Zinter *et al* [1] to determine pathogenic microorganisms in samples based on the abundance and zscore of microorganisms, and modified their method as follows: first calculate the rpm value of each detected microorganism in each sample; then count the zscore of the detected microorganisms in the sample, as follows: first normalize each rpm value; Then, the log10 (rpm) of all the samples was calculated based on the log10 (rpm) of the microorganisms detected in the sample and the log10 (rpm) of all the samples, and finally, the log10 (rpm) of the microorganisms in the sample was calculated based on the log10 (rpm) of the microorganisms in the sample (after normalisation) and the log10 (rpm) of the other samples. The zscore of the microorganism was counted according to the log10 (rpm) value of the microorganism in the sample and the log10 (rpm) value of the other samples, with the formula zscore = (sample log10 (rpm) - mean log10 (rpm) value of all samples outside the sample) / standard deviation of log10 (rpm) of all samples outside the sample. Finally, pathogenic microorganisms were screened against a threshold value, using a threshold value of rpm >= 10 and zscore >= 2.

**Reference**

1. Zinter, M.S., et al., *Pulmonary Metagenomic Sequencing Suggests Missed Infections in Immunocompromised Children.* Clin Infect Dis, 2019. **68**(11): p. 1847-1855.

**Supplementary Figures**


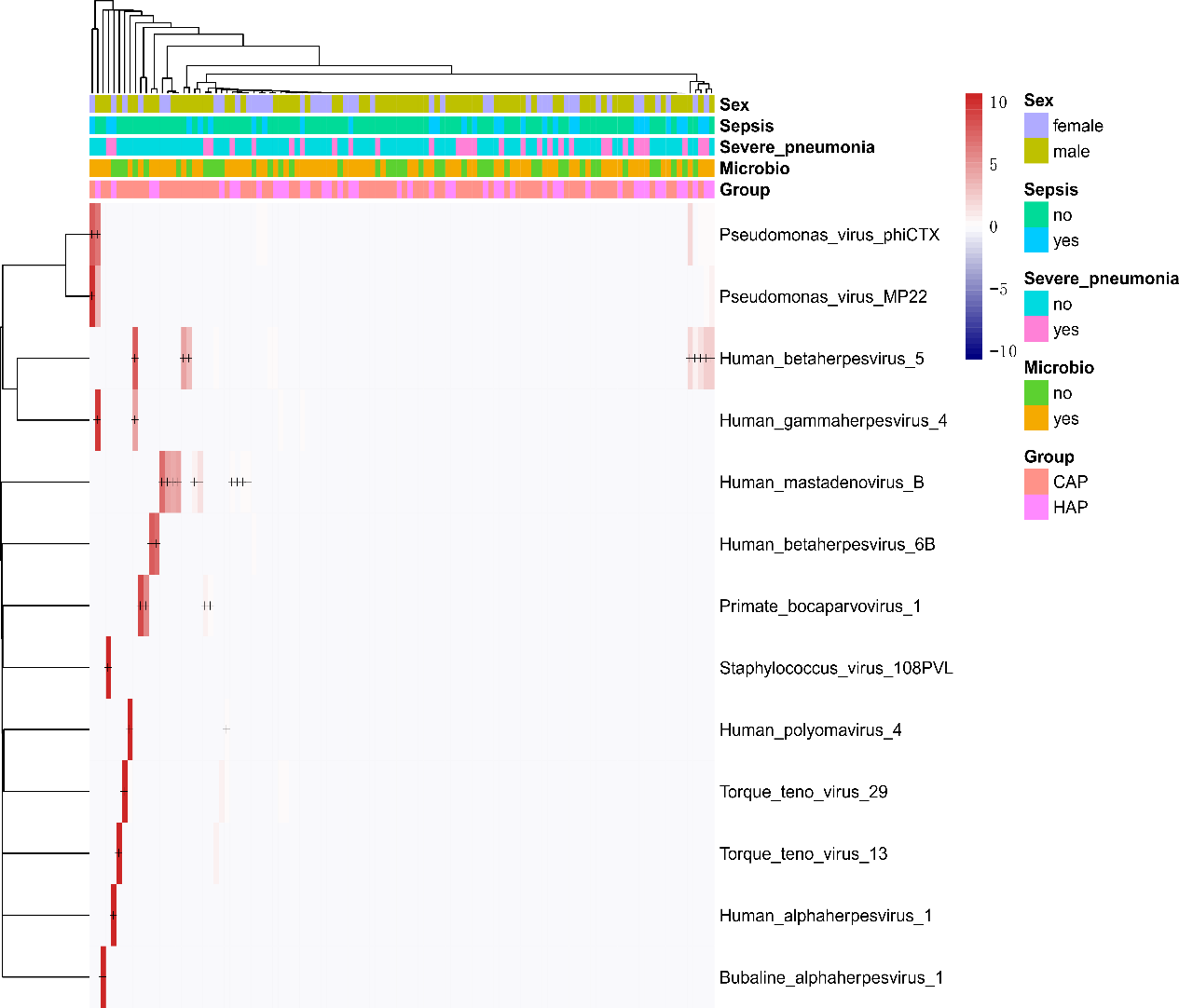


**Supplementary Figure 1. Heat map of potential viruses** **detected by mNGS of BALF samples**. Each row horizontally represents a sample, vertically represents the potential virus, the "+" and "-" signs in the graph represent significant positive and negative correlations respectively, the clinical phenotype is at the top and the colour block on the right represents the value of the specific clinical phenotype.

**
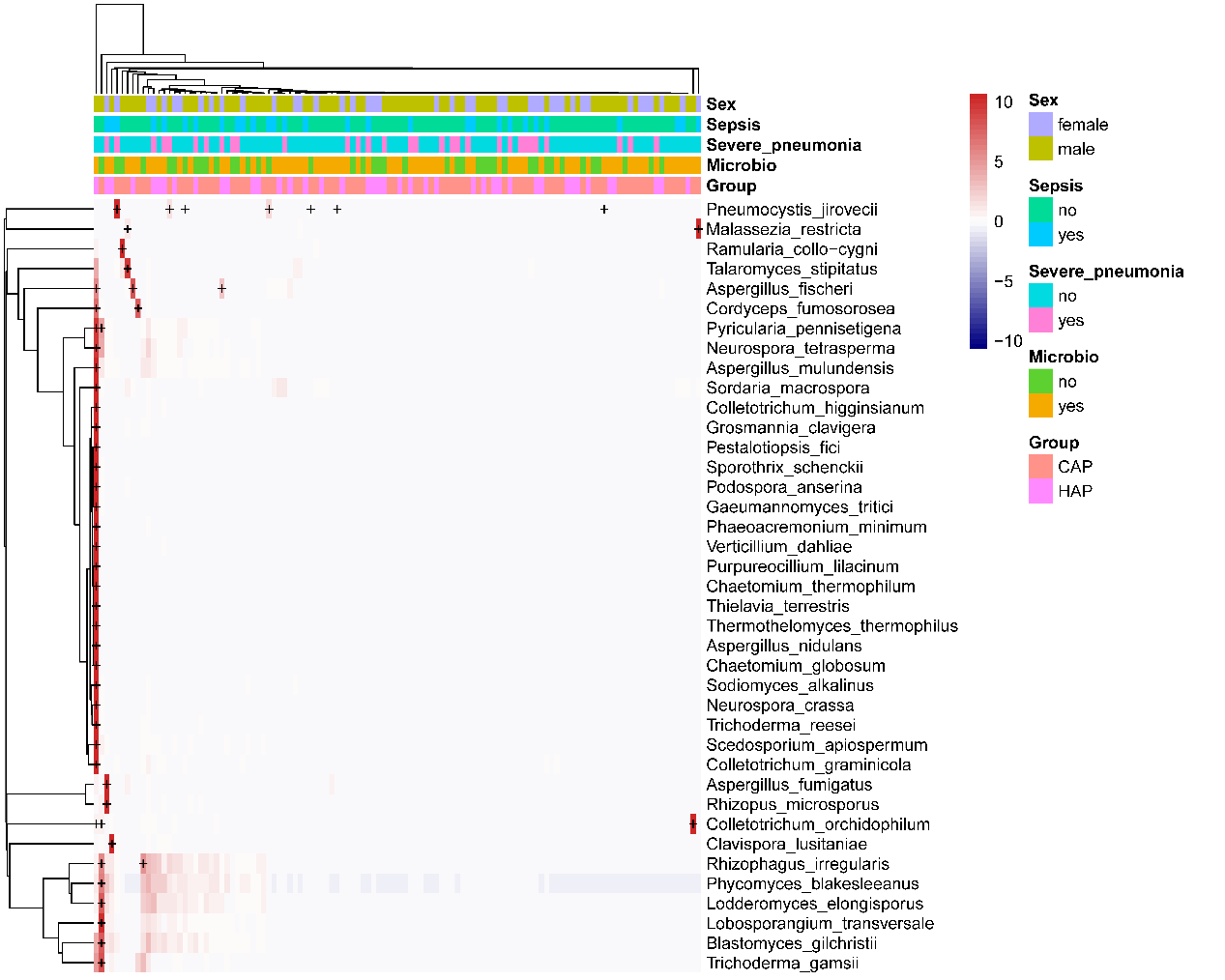
**

**Supplementary Figure 2. Heat map of potential fungi** **detected by mNGS of BALF samples**. Each row horizontally represents a sample, vertically represents the potential fungi, the "+" and "-" signs in the graph represent significant positive and negative correlations respectively, the clinical phenotype is at the top and the colour block on the right represents the value of the specific clinical phenotype.

**
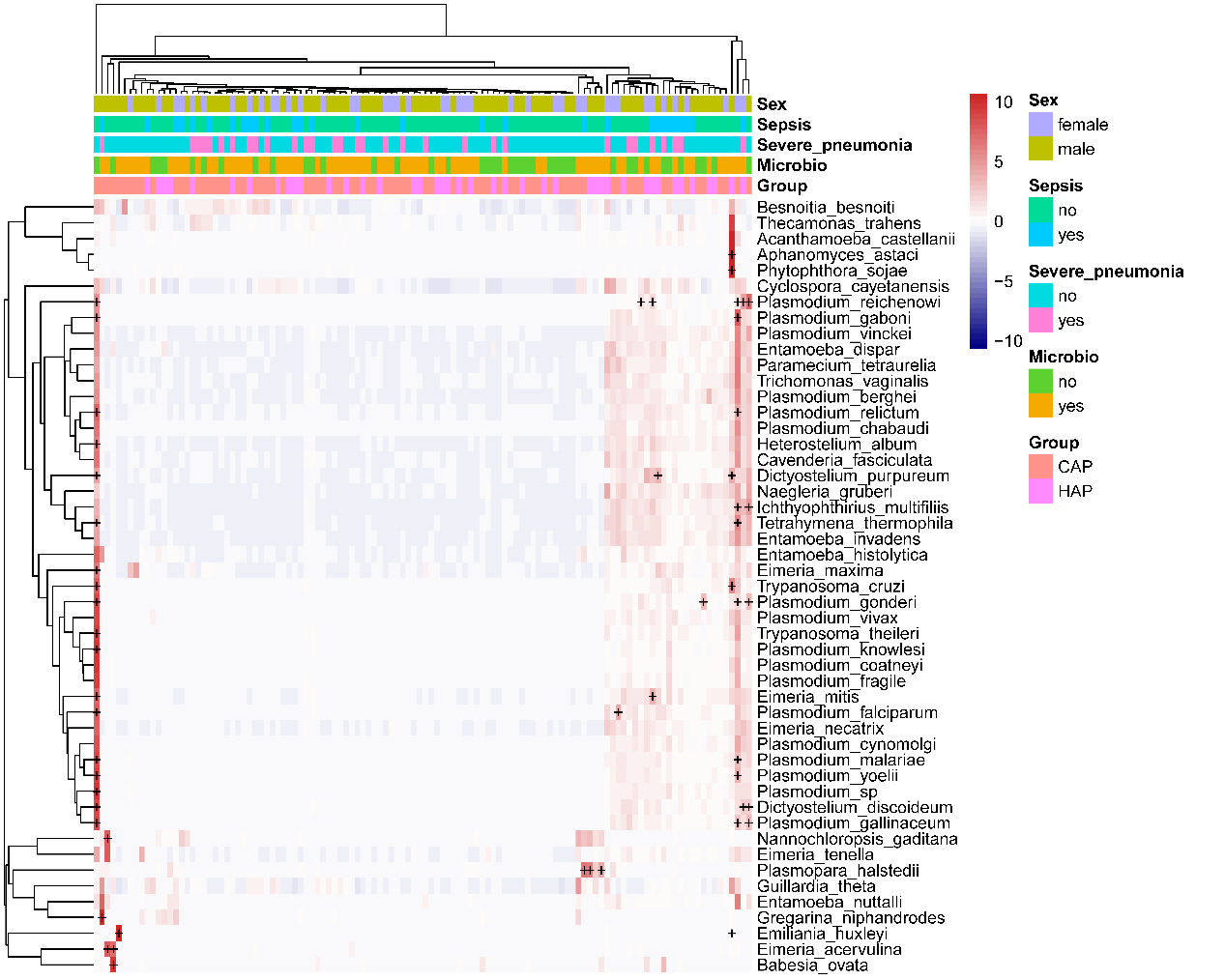
**

**Supplementary Figure 3. Heat map of potential protozoans** **detected by mNGS of BALF samples**. Each row horizontally represents a sample, vertically represents the potential **protozoa**, the "+" and "-" signs in the graph represent significant positive and negative correlations respectively, the clinical phenotype is at the top and the colour block on the right represents the value of the specific clinical phenotype.


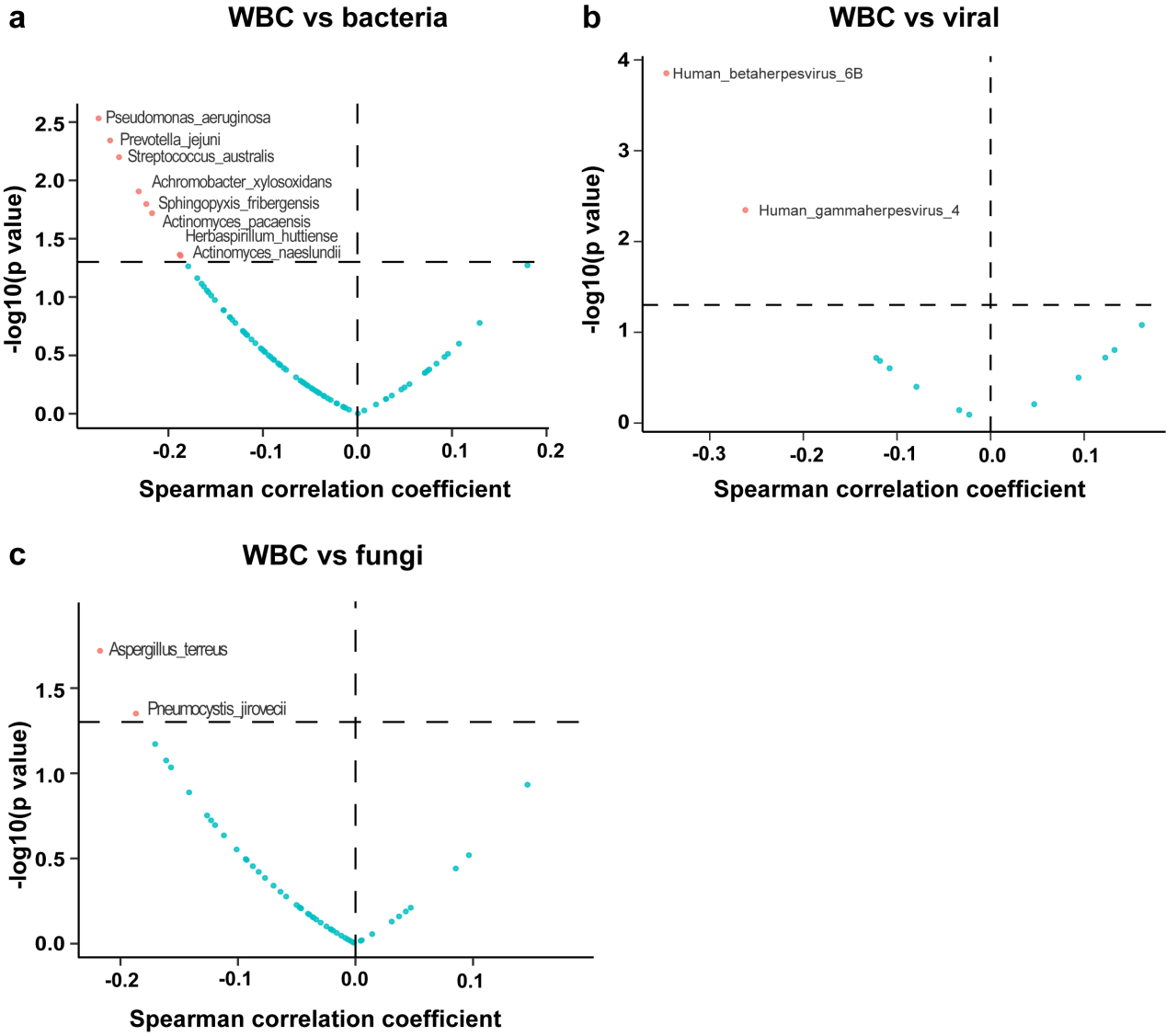


**Supplementary Figure 4. Potential pathogenic microorganisms of bronchoalveolar lavage fluid correlated with blood white blood cell count (WBC)**. Heat map display of correlation results between potential pathogenic bacteria (**A**), viruses (**B**), and fungi (**C**) of alveolar lavage fluid and blood white blood cell count (WBC), with the horizontal coordinate representing the correlation coefficient (Spearman correlation coefficient) and the vertical coordinate the negative log of the p-value of the correlation between clinical phenotype and potential pathogen, i.e. - log10(pvalue), each point in the plot represents a potential pathogenic microorganism, and screened pathogens with significant differences have been marked in red.


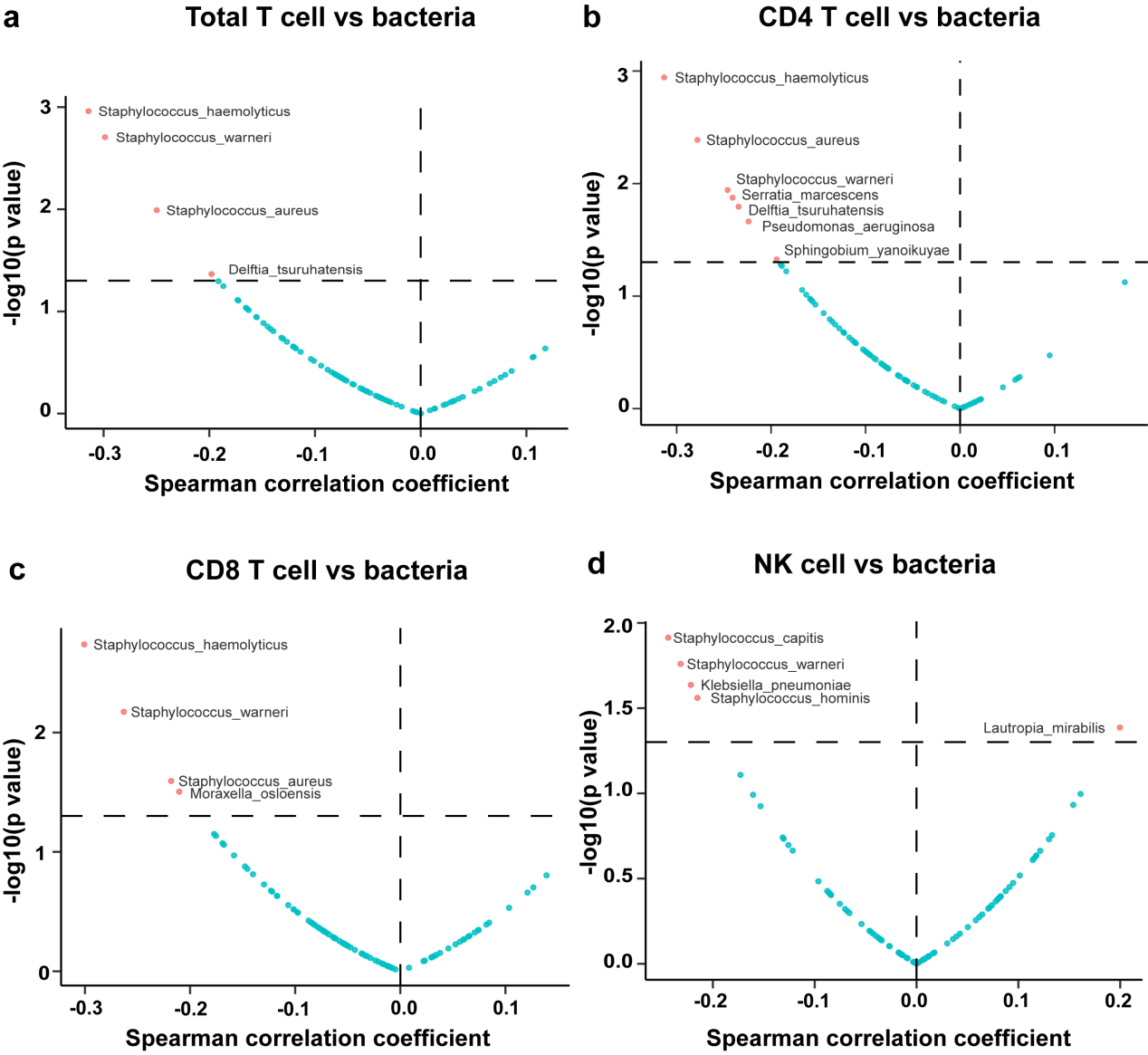


**Supplementary Figure 5. Bronchoalveolar lavage fluid pathogenic bacterial abundance correlated with T-cell counts.** Heat map display of correlation results between potential pathogenic bacteria and blood total T (**A**), CD4^+^ T (**B**), CD8^+^ T (**C**), and NK (**D**) cell counts in bronchoalveolar lavage fluid, with the horizontal coordinate representing the correlation coefficient (Spearman correlation coefficient) and the vertical coordinate the negative logarithm of the p-value of the correlation between clinical phenotype and potential pathogen Each point in the plot represents a potential pathogenic microorganism, and the pathogens screened for significant differences have been marked in red.


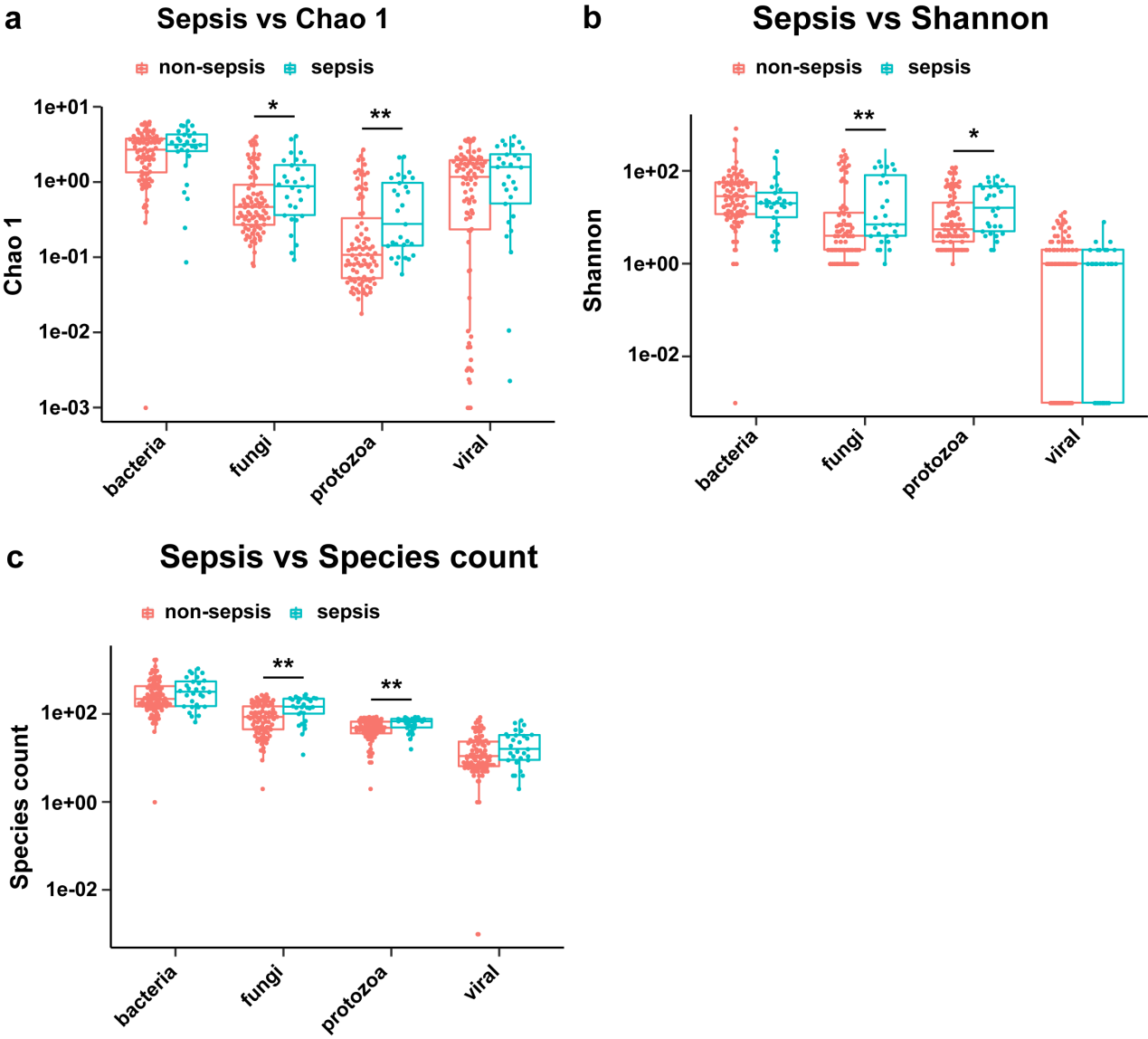


**Supplementary Figure 6. Sepsis in patients with severe pneumonia is associated with elevated Bronchoalveolar lavage fluid fungal and parasite diversity.** **a**, Chao 1; **b**, Shannon diversity; **c**, species count.


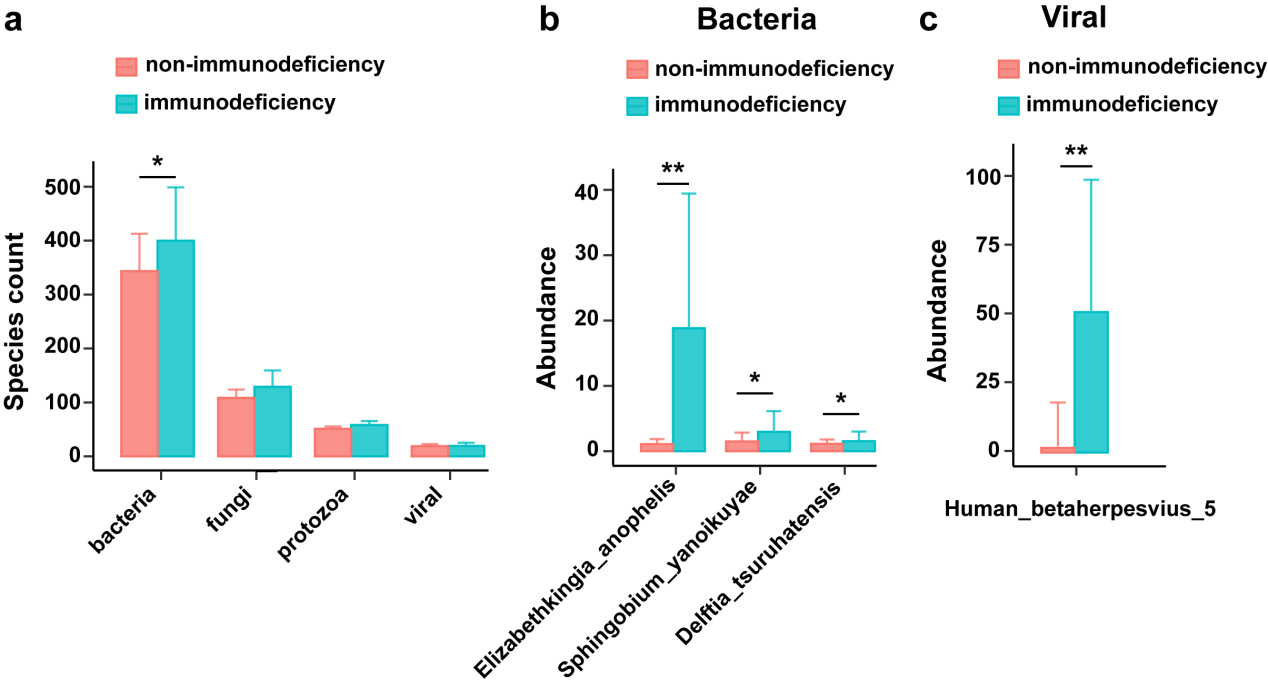


**Supplementary Figure 7. Association between immunodeficiency and** **bronchoalveolar lavage fluid pathogenic microorganisms in patients with severe pneumonia**. a, Immunodeficiency vs bronchoalveolar lavage fluid Species count relationship; b, Immunodeficiency vs bronchoalveolar lavage fluid bacteria; c, bronchoalveolar lavage fluid vs alveolar lavage fluid viruses.
